# Evaluation of the walk-through inflatable colon as a colorectal cancer education tool: results from a pre and post research design

**DOI:** 10.1186/1471-2407-14-626

**Published:** 2014-08-28

**Authors:** Janeth I Sanchez, Rebecca Palacios, Adrianna Cole, Mary A O’Connell

**Affiliations:** Plant and Environmental Sciences, New Mexico State University, Las Cruces, NM 88003 USA; Department of Public Health Sciences, New Mexico State University, Las Cruces, NM 88003 USA

**Keywords:** Colorectal cancer, Educational tools, Health knowledge, Attitudes, Practice, Hispanic Americans, Screening, Health education

## Abstract

**Background:**

Colorectal cancer (CRC) is a disease that can be prevented through early detection. Through the use of effective educational tools, individuals can become better informed about CRC and understand the importance of screening and early detection. The walk through Inflatable Colon is an innovative educational resource developed to engage and educate communities on CRC and the importance of receiving screening at the appropriate ages.

**Methods:**

The Inflatable Colon Assessment Survey (ICAS) assessed knowledge and behavioral intentions to obtain screening and promote CRC awareness. New Mexico State University faculty, staff, and students completed a consent form, took the pre-ICAS, toured the Inflatable Colon, and completed the post-ICAS. The majority of participants (92%) were young adults, mostly college students, under the age of 30 yrs.

**Results:**

Overall, participants demonstrated increases in CRC knowledge and awareness after touring the inflatable colon (p-values < 0.001). Interestingly, both males and Hispanics had lower CRC awareness at pre-test, but exhibited maximum awareness gains equal to that of females and non Hispanic Whites after touring the IC. Behavioral intentions to obtain CRC screening in the future and to promote CRC awareness also increased (p-value < 0.001). Gender differences in behavioral intentions to act as advocators for CRC education were found (p < 0.05), with females being more likely to educate others about CRC than males.

**Conclusion:**

Educational efforts conducted in early adulthood may serve to promote healthier lifestyles (e.g., physical activity, healthy nutrition, screening). These educated young adults may also serve to disseminate CRC information to high-risk friends and relatives. The walk through Inflatable Colon can increase CRC knowledge and intentions to get screened among a young and diverse population.

**Electronic supplementary material:**

The online version of this article (doi:10.1186/1471-2407-14-626) contains supplementary material, which is available to authorized users.

## Background

Colorectal cancer (CRC) is a chronic condition that can be successfully treated if detected early. In fact, significant declines in CRC mortality have been observed over the past decades [1–4], declines largely attributed to advances in CRC screening tests and treatment [5, 6]. In spite of these advancements, CRC continues to be the second leading cause of cancer related deaths among men and women in the US [1, 4]. Furthermore, the cost of treatment for CRC in the US was estimated at $14.1 billion in 2010 [7], and is projected to reach over $17 billion by 2020 [7–10].

With high incidence and mortality rates of CRC in the US, as well as high treatment costs, it is imperative to start placing a greater emphasis on CRC prevention efforts. Knowledge and awareness of CRC in the general population is low and is routinely reported as a significant barrier to compliance for CRC screening, especially among underserved populations [11–16]. The U.S. Preventive Services Task Force (USPSTF) [17] recommends starting CRC screening at 50 years. CRC prevention education, is often coupled with efforts to promote such screening among individuals in this age group. Recent studies, however, suggest CRC prevention education needs to start occurring much earlier than CRC screening promotion efforts. For example, increasing trends in CRC incidence among individuals younger than 50 years, especially among those younger than 40 years of age [18, 19] point to the need for CRC prevention education in young adulthood. Specifically, Siegel and colleagues [19] found that relative to adults 50 years and older who demonstrated a 1.8% annual decrease in CRC incidence, young adults between the ages of 20 and 29 years demonstrated the highest annual percent increase in CRC incidence (5.2% for men and 5.6% for women). These increasing CRC trends in young adults mirror increasing trends toward greater obesity and other CRC risk factors in the U.S. [19, 20]. Thus, while standard CRC screening is not recommended for young adults, CRC prevention education starting in early adulthood may be beneficial in reducing CRC risk factors and reversing increasing trends of CRC incidence in young adulthood [21].

Gender and ethnic disparities in CRC incidence among young adults have been reported. Specifically, Siegel and colleagues [19] found that increases in CRC incidence among individuals younger than 50 years were not equal across ethnic and gender groups. Compared to non-Hispanic White (NHW) males, Hispanic males demonstrated higher increases in CRC incidence (2.0% vs. 2.7%). When analyzing gender by ethnicity patterns, this study found that NHW women had greater increases in CRC incidence than NHW men (2.2% for women and 2.0% for men); however, this pattern was reversed and more extreme for Hispanics (1.1% for women and 2.7% for men). Meyer et al. [18] also identified racial and ethnic differences in CRC incidence. While all groups younger than 40 years demonstrated increases in rectal cancer, Whites (2.5%) demonstrated greater increases than Blacks (1.9%). This research highlights the importance of examining interactions in health outcomes by gender and ethnicity and ensuring that cancer prevention outreach efforts are properly engaging gender and ethnic subgroups that are at greater risk for CRC.

The challenge to promoting healthy lifestyles, however, lies in designing effective interventions for the general public. Public health interventions that include visual tools in combination with text or audio text are more effective at increasing knowledge, comprehension, and retention when compared to text only materials [22–24]. An additional advantage of these communication tools is that they are effective in educating populations with low levels of health literacy, a characteristic associated with adverse health outcomes [24–27].

**Figure 1:**
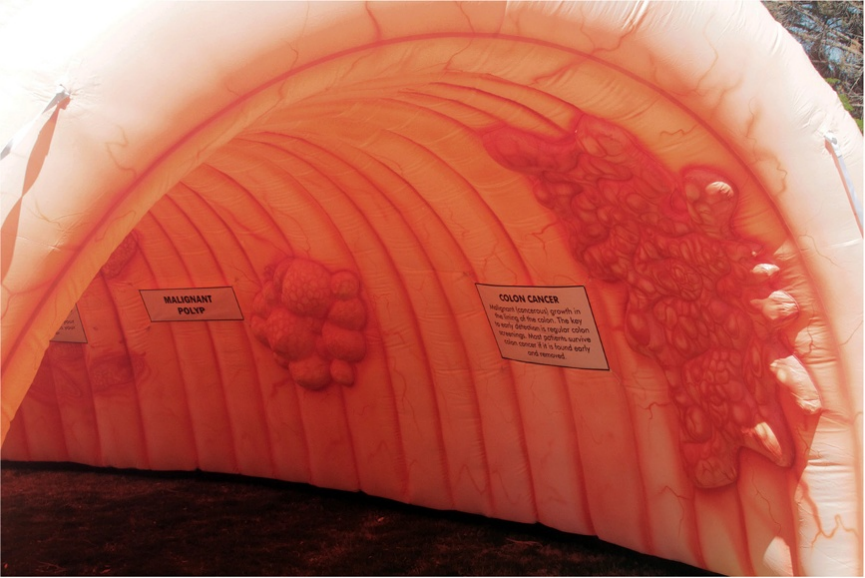
**Walk through Inflatable Colon.**

The inflatable colon (IC) is an innovative, visual, and interactive educational resource designed to engage and educate communities at risk for CRC (Figure 1). To date only one study has examined the effectiveness of the IC [28]. Specifically, this study identified significant gains for knowledge, intentions to obtain screening, and social support among Alaskans who toured the IC [28]. Based on these promising outcomes, the effectiveness of the IC as an interactive CRC educational tool and evidence-based practice should be further examined in diverse populations.

The purpose of the present study was to assess the effectiveness of the IC as a CRC educational tool among a young and diverse population. Specifically, this study examined increases in CRC knowledge, awareness, and behavioral intentions to obtain CRC screening and to promote CRC education after touring the IC. Gender and ethnic differences in study outcomes were also determined.

## Methods

### Ethics

This study involved human subjects and was performed only after review and approval. The New Mexico State University Institutional Review Board (FWA00000451) approved all study procedures and the survey instruments (NMSU IRB approval #7385). Written informed consent prior to participation was obtained from all participants: members of a focus group or study participants in the Inflatable Colon Assessment Survey.

### Participants

New Mexico State University faculty, staff, and students were invited to participate in the present study. College students were recruited to participate in the study through their classes and university newsletters. A total of 23 professors agreed to provide extra credit for their students participating in the study. A list of participants was given to each professor who agreed to provide extra credit. Flyers around campus informed staff and faculty on the availability of the IC on campus.

### Inflatable colon

The Inflatable Colon (IC) is a walk-through innovative and theory-based educational tool for CRC (Figure 1). The IC is 20 × 15 × 10 feet (l × h × w) and depicts 6 different precursors and stages of CRC: normal colon tissue, Crohn’s disease, polyps, malignant polyps, colon cancer, and advanced colon cancer. The signage includes the title of each condition along with a brief description in both English and Spanish. The Cognitive Theory of Multimedia Learning and the Three Principles of Perceptions, which include Figure/Ground Perceptions, Hierarchy Perceptions, and Gestalt Perceptions [29], were used to develop the IC educational tool. The IC depicts how CRC may progress if it is not detected early and demonstrates certain risk factors that may increase an individual’s risk of developing CRC.

### Procedure

The IC was set-up for five days in March 2012 (CRC Awareness Month) at various locations throughout the NMSU campus. Participants completed a consent form and the pre-ICAS followed by a tour of the IC conducted by three different tour leaders, the National Outreach Network’s Community Health Educator and two research assistants. In order to promote consistency in program delivery, all tour leaders were trained to cover a standard list of educational points during the tour. Specifically, the tour included information regarding CRC, its risk factors (e.g. physical activity, nutrition, genetics), stages of CRC, and CRC screening methods (fecal occult blood test, sigmoidoscopy, and colonoscopy). The IC tour also informs participants on the USPSTF recommendations to obtain CRC screening starting at 50 years [17]. Although the tours were available in Spanish, all participants requested tours in English. The tour took approximately 10 to 15 minutes to complete with no more than 10 people at a time. After the tour, participants were asked to complete the post-ICAS. Colorectal Cancer educational materials (e.g. brochures, booklets, handouts, etc.) were available for participants after completion of the IC study.

### Instruments

The Inflatable Colon Assessment Survey (ICAS) a pre- and post-test, was developed to evaluate CRC knowledge (i.e., what the person actually knows about CRC) and CRC awareness (i.e., what the person has heard about CRC). This instrument was also designed to evaluate behavioral intentions to obtain CRC screening and intentions to disseminate or promote CRC health information to family members, peers and community members. A pdf version of this survey instrument is provided as Additional file 1. All questions were reviewed by community members for clarity and content.

The pre-ICAS included a total of 36 items: 2 items assessed prior CRC education or prior touring of the IC, 8 awareness and 5 knowledge items, 7 behavioral intention items, and 14 individual items assessing demographics, regular sources of health care, and physician recommendations to obtain CRC screening. The pre-ICAS CRC awareness and knowledge questions consisted of yes or nor responses and were adapted from published tools on CRC knowledge and awareness, attitudes, beliefs and screening [30–32]. The post-ICAS contained a total of 33 items (Table 1). In addition to CRC awareness, knowledge and behavioral intentions items, the post-ICAS included items on behavioral intentions to encourage others to tour the IC, the likelihood of the IC being accepted in their culture as an educational tool, and perceptions of the IC as an effective CRC educational tool. The pre- and post-ICAS, each took approximately 12 to 15 minutes to complete.

**Table 1:** **Colorectal cancer awareness, knowledge, and behavioral intentions items**

Category	Survey question
**Awareness items**	Do you know what colorectal cancer is?
	Do you know what a colon polyp is?
	Do you know what a cancer screening test is?
	Do you know the different types of screening tests available for colorectal cancer?
	Do you know what the following tests are:
	Fecal Occult Blood Test (FOBT)/ Stool Blood Test?
	Colonoscopy?
	Sigmoidoscopy?
	Do you know where you can obtain screening tests for colorectal cancer?
**Knowledge items**	Do you think a diet low in fat and high in fiber helps decrease the risk for developing colorectal cancer?
	Do you think physical activity decreases the risk of developing colorectal cancer?
	Do you think the risks for developing colorectal cancer increases after the age of 50?
	Do you think most patients survive colorectal cancer if it is found early and removed?
	Do you think you ONLY need colorectal cancer screening if you are having any symptoms?
**Behavioral intention to obtain colorectal cancer screening**	Do you plan on talking to your doctor about cancer of the colon and rectum in the future?
	Do you plan on getting screened for cancer of the colon and rectum in the future?
**Behavioral intentions to promote colorectal cancer education**	How likely are you to talk about colorectal cancer with your:
	Parents
	Grandparents
	Relatives (aunts, uncles, cousins)
	Peers (friends, colleagues, etc.)
	Community members
	Individuals at risk (50+ years of age, family history, etc.)

The Flesch-Kincaid Grade Level Scale was utilized to evaluate the readability of the materials. The pre-ICAS measured at a 7^th^ grade level while the post-ICAS measured at a 9^th^ grade level; the consent form measured at a 12^th^ grade reading level and the signage of the inflatable colon measured at an 8^th^ grade level. The readability level of all instruments was appropriate for the college population participating in this study.

### Data analysis and reduction

Composite scores were developed for conceptually related items, including CRC knowledge (sum of eight items, possible range of scores 0 to 8), CRC awareness (sum of five items, possible range of scores 0 to 5), and behavioral intentions to promote CRC education (mean of six items). Statistical Package for the Social Sciences (SPSS) Version 20.0 was used to conduct the analysis; multivariate analysis of variance (MANOVA) was used to examine between (gender and ethnicity) and within subjects (pre- and post-test) program effects. Age was not included as a between subjects factor because the majority of participants (88%) were less than 30 years of age. Only 3 (<1%) were 50 years and older.

## Results

### Participant characteristics

#### Demographics

A total of 485 NMSU faculty, staff, and students completed the IC tour and the ICAS tests; of these only 22 (4.5%) had previously taken a tour of the inflatable colon prior to participating in this study. These individuals were removed from further analysis, resulting in a sample size of 463 individuals.

The participants were predominantly female (67%) and ages ranged between 20 to 69 years of age, with 92% aged 20 to 29 years old (Table 2). The racial/ethnic composition was predominantly Hispanic (50%), followed by 32% non-Hispanic White (NHW), 6% Black, 6% Native American, and 5% Asian. Ethnic comparisons were limited to NHW and Hispanics due to the small sample size for the other race/ethnicities in this study. Most participants, as expected, reported having some college education since the study was held at a university campus.

**Table 2:** **Demographic characteristics of participants in the inflatable colon educational intervention**

Characteristic	Total ^a^(n = 463)	Non-hispanic white (n = 149, 32.2%)	Hispanic (n = 233, 50.3%)
**Gender**			
Male	156 (33.7%)	45 (30.2%)	70 (30.0%)
Female	307 (66.3%)	104 (69.8%)	163 (70.0%)
**Age**			
20-29	426 (92.0%)	134 (89.9%)	216 (92.7%)
30-39	24 (5.2%)	11 (7.4%)	10 (4.3%)
40-49	10 (2.1%)	3 (2.0%)	5 (2.1%)
50 +	3 (0.6%)	1 (0.7%)	2 (0.8%)
**Education Level**			
12^th^ grade or less	6 (1.2%)	6 (4.0%)	3 (1.3%)
High school Graduate or GED	31 (6.7%)	5 (3.4%)	21 (9.0%)
Some college (no degree)	376 (81.2%)	124 (83.2%)	188 (80.7%)
College and advanced degrees (MA, MD, PhD, JD)	50 (10.8%)	19 (12.8%)	21 (9.0%)
**Regular Health Clinic**			
Yes	221 (47.7%)	83 (55.7%)	113 (48.5%)
No	240 (51.8%)	65 (43.6%)	119 (48.5%)
**Regular Physician**			
Yes	217 (46.9%)	76 (51.0%)	107 (45.9%)
No	239 (51.6%)	72 (48.3%)	122 (52.4%)
**Health Care Plan/Insurance**			
Private Health Insurance (self acquired)	112 (24.2%)	46 (30.9%)	44 (18.9%)
Private Health Insurance (employer acquired)	130 (28.1%)	55 (36.9%)	58 (24.9%)
Medicare	26 (5.6%)	3 (2.0%)	15 (6.4%)
Medicaid	33 (7.1%)	5 (3.4%)	22 (9.4%)
Veteran’s Affairs Health Insurance (VA)	11 (2.4%)	3 (2.0%)	5 (2.1%)
Other	39 (8.4%)	9 (6.0%)	17 (7.3%)
None	109 (23.5%)	27 (18.1%)	70 (30.0%)

^a^includes individuals who did not self-identify as Hispanic or non-Hispanic white; (black, 27, Native American, 29, Asian/Pacific Islander, 18, and other, 7).

#### Usual care (clinic & doctor)

Among the participants, 47% reported having a regular doctor and 48% stated having a regular source of health care. The university campus health center served as the source of healthcare for one fifth of the sample.

#### Insurance coverage

Seventy six percent of participants reported some type of insurance coverage. Of these, 28% were insured through employer-based private health insurance, 24% had self-paid private health insurance, and 15% relied on publicly funded insurance (e.g., Medicare, Medicaid, and Veterans Affairs).

#### Doctor referral for CRC screening

Only a small number of the participants had a physician recommend them to obtain CRC screening (6%). Among the participants who had been referred to obtain a colonoscopy, 62% were in the 20 to 29 age groups, 23% were in the 30 to 39 age group, and 15% were 40 years of age and older.

### CRC knowledge and awareness

A three-way MANOVA with Gender (male, female) and Race/Ethnicity (NHW and Hispanic) as the between subjects factors and Time (pre, post) as the sole within subjects factor was conducted using CRC Knowledge and CRC Awareness as the dependent variables. The results of this analysis revealed significant multivariate effects for Gender, F (2,376) = 4.46, p = .01 η_p_^2^ = 0.023, Ethnicity, F (2,376) = 9.65, p < 0.001, η_p_^2^ = 0.05, and Time, F (2,376) = 821.19, p < 0.001, η_p_^2^ = 0.81, and significant interactions for Gender x Time, F (2,376) = 5.95, p = 0.003, η_p_^2^ = 0.03, and Ethnicity x Time, F (2,376) = 3.12, p = 0.05, η_p_^2^ = 0.02.

**Figure 2:**
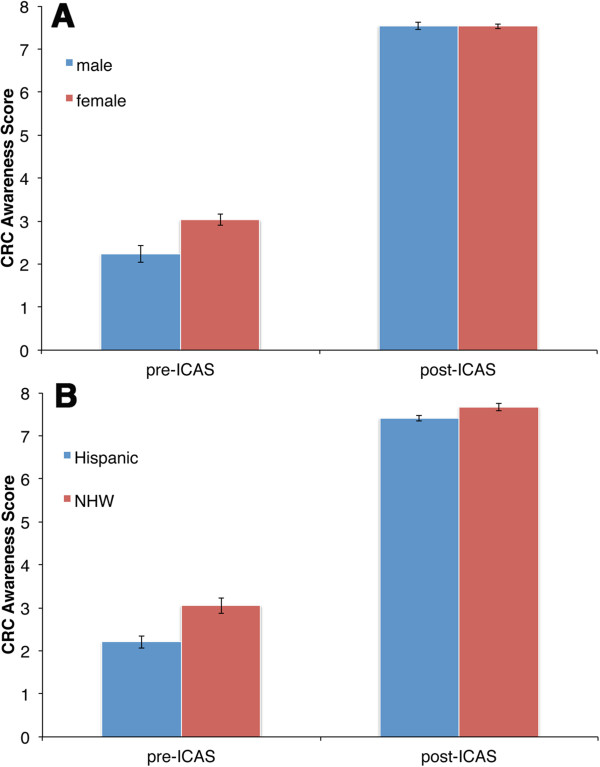
**Differences in CRC Awareness measured by pre- and post-ICAS.** The mean CRC awareness scores for the pre- and post test are shown. **A**. male (blue bars) and female (red bars) participants; **B**. Hispanic (blue bars) and non-Hispanic white (NHW) (red bars) participants. Error bars indicate standard errors.

Individual two-way univariate ANOVAs revealed a significant Time effect for both CRC Knowledge and CRC Awareness with participants demonstrating increases from pre-test in knowledge and awareness at post-test (both p values < 0.001, see Table 3). Table 4 demonstrates between-subjects effects for Gender in CRC Awareness (Male 4.89 vs. Female 5.28; p = 0.003) and for Ethnicity in CRC Knowledge (NHW 4.64 and Hispanic 4.50; p = 0.03) and CRC Awareness (NHW 5.36 and Hispanic 4.81; p values < 0.001). Significant Gender x Time (p = 0.001) and Ethnicity x Time (p = 0.02) effects were also identified for CRC Awareness (see Figure 2). As Figure 2A shows, males exhibited lower awareness than females at pre-test, but exhibited similar awareness levels at post-test. Similarly, Figure 2B shows that Hispanics exhibited lower awareness than NHWs at pre-test, but exhibited similar awareness levels at post-test.

**Table 3:** **Effect of the inflatable colon educational intervention on categories of colorectal cancer (CRC) knowledge and screening**

	Pre-ICAS ^a^	Post-ICAS	
Categories on ICAS	mean (SE)	mean (SE)	p^b^
CRC^c^ awareness	2.63 (0.12)	7.54 (0.05)	<0.001
CRC knowledge	4.39 (0.05)	4.75 (0.03)	<0.001
Intentions to obtain CRC screening	2.67 (0.07)	4.13 (0.06)	<0.001
Intentions to promote CRC education	2.69 (0.07)	3.85 (0.05)	<0.001

^a^ICAS, Inflatable Colon Assessment Survey.

^b^p values were determined using multivariate analysis of variance.

^c^CRC, colorectal cancer.

**Table 4:** **Gender and ethnic differences in categories on Inflatable Colon Assessment Survey (ICAS)**

	Male	Female		NHW ^a^	Hispanic	
Categories on ICAS	mean (SE)	mean (SE)	p^b^	mean (SE)	mean (SE)	p
CRC awareness	4.89 (0.11)	5.28 (0.07)	<0.001	5.36 (0.10)	4.81 (0.08)	<0.001
CRC knowledge	4.54 (0.05)	4.60 (0.03)	0.36	4.64 (0.05)	4.50 (0.04)	0.03
Intentions to obtain CRC screening	3.39 (0.09)	3.41 (0.06)	0.86	3.44 (0.08)	3.36 (0.06)	0.44
Intentions to promote CRC education	3.15 (0.08)	3.40 (0.06)	0.01	3.22 (0.08)	3.32 (0.06)	0.32

^a^NHW, non-Hispanic white; ^b^p values were determined using multivariate analysis of variance.

### Behavioral intentions

A three-way MANOVA with Gender (male, female) and Race/Ethnicity (NHW and Hispanic) as the between subjects factors and time (pre, post) as the sole within subjects factor was conducted using Behavioral Intentions to Obtain Screening and Behavioral Intentions to Promote CRC Education as the dependent variables. The results of this analysis revealed significant multivariate effects for Time, F (2,368) = 264.73, p < 0.001, η_p_^2^ = 0.59, and Gender, F (2,368) = 4.03, p = 0.02, η_p_^2^ = 0.02.

Individual two-way univariate ANOVAs revealed a significant Time effect for both Behavioral Intentions to Obtain Screening and Behavioral Intentions to Promote CRC Education (both p values < 0.001), with study participants demonstrating greater behavioral intentions at post-test relative to pre-test (see Table 3). A between subjects effect in Gender was identified for Behavioral Intentions to Promote CRC Education (Male 3.15 and Female 3.40; p = 0.01, see Table 4).

### Perceived effectiveness of IC and cultural acceptance of IC

Overall, study participants perceived the IC to be an effective tool to educate individuals about CRC at time of post-ICAS (Mean: 4.62 on a 5 point scale). Participants also responded that the IC was likely to be accepted in their culture as an educational tool for CRC (Mean: 4.28 on a 5 point scale).

A two-way MANOVA with Gender (male, female) and Race/Ethnicity (NHW and Hispanic) as the between subjects factors and Perceived Effectiveness of IC and Cultural Acceptance of IC as the dependent variables was conducted. The results of this analysis revealed a significant multivariate Gender effect, F (2,391) = 5.78, p = 0.01, η_p_^2^ = 0.02. A between subjects effect in Gender was identified for both Perceived Effectiveness of IC and Cultural Acceptance of IC, with females rating greater perceived effectiveness (Male 4.53 vs. Female 4.64; p = 0.05) and cultural acceptance of the IC than males (Male 4.11 vs. Female 4.39; p < 0.007).

## Discussion

The USPSTF screening guidelines recommend that CRC screening start at 50 years of age [17]. Past interventions promoting these CRC screening guidelines have included educational components to enhance CRC knowledge and awareness. CRC education is essential as knowledge and awareness in the general population is low [14]; 80% of primary care physicians consider this the most important barrier to compliance for CRC screening [11]. Research designed to determine barriers to CRC screening compliance among underserved groups identify lack of knowledge and awareness as persistent themes [12, 13, 16]. In most of these studies, the interventions or participant group have focused on educating older adults of screening age (50 years and older). By this age individuals may have already engaged in a lifetime of unhealthy practices predisposing them to CRC. They may also have gone through life unaware of their genetic predisposition for CRC.

The present study found that all participants who had been physician-referred for a colonoscopy were younger than 50 years of age. Although the reasons why these adults were referred for CRC screening were not assessed in this study, we can speculate that physicians may be identifying significant genetic or biological CRC precursors to warrant screening referrals at earlier ages than 50. Specifically, USPSTF screening guidelines recommend that adults younger than 50 years presenting with biological risk factors, such as CRC family history or Crohn’s disease, may benefit from CRC screening at earlier ages [17]. In addition to biological precursors, physicians may be identifying additional behavior risk factors, which have shown a relationship to CRC such as obesity and smoking.

Meyer and colleagues, found that individuals younger than 40 years of age are exhibiting increases in rectal cancer but not colon cancer [18]; others have demonstrated a 1.5% increase in CRC incidence among young adults (<50 years) from 1992 to 2005 [19]. These studies, as well as the present study, suggest that educational efforts are needed in early adulthood to increase awareness of biological risk factors for CRC and to promote a healthier lifestyle (e.g., physical activity, healthy nutrition, timely screening for high risk individuals), which may lead to a reduced risk of developing CRC over one’s lifespan [21, 33, 34]. Such efforts may also help to reverse increasing CRC trends identified in young adults [19]. Finally, it is important to note, that these findings do not suggest that all young adults should be screened regularly, but only those considered by a physician to warrant early CRC screening prior to the CRC recommended screening age of 50 years.

The channel through which CRC information is disseminated should be theory-based and tailored to the varying ages and ethnicity of the audience/participants [35]. In addition, such efforts should demonstrate effective ways to communicate with one’s healthcare provider [36] especially since underserved minority populations have lower screening rates [37] and are less likely to be referred for CRC screening [2]. Although text only materials have been the typical channel for disseminating cancer health education [38] recent innovative tools have been designed to incorporate audiovisual stimuli and be more interactive [22, 28, 33]. The IC is one such innovative tool that has been incorporated into programs to educate diverse populations about CRC and the benefits of screening [39, 40]. However, to date, only one program based in Alaska [28] has reported an IC’s effectiveness; using a pre-post test design, touring the IC significantly improved CRC knowledge, intention to get screened and comfort about talking about CRC with others. These results are similar to the results presented here in this study, where the ICAS demonstrated gains in CRC knowledge, intention for screening and intention to promote CRC screening (Table 3, Figure 2). The populations in these two studies were quite different; in the Alaskan study, 31% were under age 35, 37% were Alaskan Natives/American Indian/Aboriginal Canadian and 71% were female. In contrast, the participants in the present study (Table 2) are predominantly under age 30 and Hispanic. While the sample population in this current study was a convenience sample, it was ethnically representative of the state of New Mexico. Across these very diverse populations in either Alaska or New Mexico, the IC was an effective educational tool.

Overall, participants in this study demonstrated an increase in CRC knowledge and awareness after touring the IC, including the importance of physical activity and good nutrition for decreasing one’s CRC risk. The gains in CRC awareness were notable; the scores increased 186%. Comparing the effectiveness of this intervention for improved CRC awareness or knowledge with other intervention methods is difficult as there is no shared pre- post-test. However, Meade et al. [23, 32] reports 23-26% score improvements following a CRC educational session using booklets or videotapes, and Hart et al. [41] using leaflets doubled the number of individuals with correct responses.

Interestingly, both men and Hispanics started off with lower CRC awareness at pre-test, but exhibited maximum awareness gains equal to that of women and NHWs after touring the IC (Figure 2). This suggests that the IC educational tool was effective with groups of different literacy or awareness levels at pre-test. This is significant particularly when one considers that both men and Hispanics experience CRC disparities in incidence and/or mortality [18, 19, 22, 42].

Following the IC tour, young adults in this study reported increased intentions to get screened for CRC in the future. Importantly, they also demonstrated increased behavioral intentions to promote CRC education among family members, peers and community members after touring the IC. Since social ties may have a large influence on changing health behaviors [34] educated young adults may serve as effective channels through which CRC information can be disseminated to high-risk family members, friends, and community members.

In addition, gender differences in behavioral intentions to act as advocators for CRC education were found, with females being more likely to educate others about CRC than males. This may reflect the role of women as health advocates for their families and community [43]. The present study also found that participants perceived the IC to be an effective and culturally acceptable CRC educational tool with females rating the IC to be more effective and culturally acceptable than males. Since women adopt the role of health advocates in society, their acceptance of the IC as an effective and culturally acceptable educational tool is an important result.

### Limitations

There were some limitations to the present study. The sample of participants who were older than 40 and 50 years of age was too small to permit any age group analyses on intentions to get screened. Future studies examining college populations should actively recruit faculty and staff in these age ranges to participate. Another limitation of this study was that it did not assess the reasons why participants younger than 50 years were referred for CRC screening or whether they were at increased risk for developing CRC. Future studies assessing whether individuals have been screened for CRC, should also assess the reasons leading to the screening referrals and individual risk factors for CRC. An additional limitation consisted of our inability to determine whether reported behavioral intentions to get screened for CRC by the young adult sample actually translated into behavioral outcomes (CRC screening later in life). Future studies assessing behavioral intentions in young adulthood would benefit from a longitudinal research design. Another limitation was attributed to the majority of the participants in this study being college students who received extra credit for their participation by university professors. This might have increased response bias if participants felt the need to respond in a socially desirable manner in order to obtain their extra credit. In order to minimize such bias, consents forms were designed to assure confidentiality of students’ response. Additional limitations included the self-report format and the lack of measures assessing behavioral intentions to engage in a healthier lifestyle.

## Conclusion

This study examined the effectiveness of the IC as a new and innovative CRC educational tool. With cancer surveillance systems demonstrating increased incidence of CRC at younger ages, this study demonstrated that the IC can be an effective educational tool for increasing CRC knowledge, awareness and behavioral intentions to get screened among a diverse population of young adults. More specifically, the IC tool can be used to educate young adults on a healthier lifestyle for reducing their CRC risk, including increasing physical activity, fruit and vegetable consumption, and consuming a high fiber diet. Furthermore, use of the IC educational tool with young adults may actually facilitate the dissemination of CRC information, as we also saw an increase in intention to promote CRC education following the intervention. Given the popularity of the IC at community events and its ability to engage the public in CRC awareness and education, future research should continue to examine its effectiveness as an educational tool among at-risk and diverse populations, particularly in longitudinal studies examining CRC behavioral and health outcomes. Finally, such research would benefit from more thorough assessment of 1) population CRC risk factors, 2) prevalence and reasons for doctor referrals to CRC screening in young adults, and 3) CRC screening behavioral outcomes.

## Electronic supplementary material

Additional file 1: **Pre- and Post-ICAS.** (PDF 704 KB)

## Abbreviations

CRC: Colorectal cancer
IC: Inflatable colon
ICAS: Inflatable colon assessment survey
MANOVA: Multivariate analysis of variance
NHW: Non-hispanic white
NMSU: New Mexico State University
SPSS: Statistical Package for the Social Sciences
USPSTF: U.S. Preventive Services Task Force.

## References

[1] American Cancer Society (2013). Cancer Facts & Figures 2013.

[2] Sanchez JI, Palacios R, Thompson B, Martinez V, O’Connell MA (2013). Assessing Colorectal Cancer Screening Behaviors and Knowledge among At-Risk Hispanics in Southern New Mexico. J Cancer Ther.

[3] Naishadham D, Lansdorp-Vogelaar I, Siegel R, Cokkinides V, Jemal A (2011). State disparities in colorectal cancer mortality patterns in the United States. Cancer Epidemiol Biomarkers Prev.

[4] Richardson L, Tai E, Rim S, Joseph D, Plescia M (2011). Vital Signs: Colorectal Cancer Screening, Incidence, and Mortality — United States, 2002–2010. MMWR Morb Mortal Wkly Rep.

[5] Edwards BK, Ward E, Kohler BA, Eheman C, Zauber AG, Anderson RN, Jemal A, Schymura MJ, Lansdorp-Vogelaar I, Seeff LC, van Ballegooijen M, Goede SL, Ries LA (2010). Annual report to the nation on the status of cancer, 1975–2006, featuring colorectal cancer trends and impact of interventions (risk factors, screening, and treatment) to reduce future rates. Cancer.

[6] Shaukat A, Mongin S, Geisser M, Lederle F, Bond J, Mandel J, Church T (2013). Long-term mortality after screening for colorectal cancer. N Engl J Med.

[7] Mariotto AB, Yabroff KR, Shao Y, Feuer EJ, Brown ML (2011). Projections of the cost of cancer care in the United States: 2010–2020. J Natl Cancer Inst.

[8] Warren JL, Yabroff KR, Meekins A, Topor M, Lamont EB, Brown ML (2008). Evaluation of trends in the cost of initial cancer treatment. J Natl Cancer Inst.

[9] Brown M, Riley G, Schussler N, Etzioni R (2002). Estimating health care costs related to cancer treatment from SEER-Medicare data. Med Care.

[10] Ó Céilleachair A, Hanly P, Skally M, O’Neill C, Fitzpatrick P, Kapur K, Staines A, Sharp L (2013). Cost comparisons and methodological heterogeneity in cost-of-illness studies: the example of colorectal cancer. Med Care.

[11] Klabunde CN, Vernon SW, Nadel M, Breen N, Seeff LC, Brown ML (2005). Barriers to colorectal cancer screening: A comparison of reports from primary care physicians and average risk adults. Med Care.

[12] Crookes DM, Njoku O, Rodriguez MC, Mendez EI, Jandorf L (2014). Promoting Colorectal Cancer Screening through Group Education in Community-Based Settings. J Cancer Educ.

[13] Filippi MK, Braiuca S, Cully L, James AS, Choi WS, Greiner KA, Daley CM (2013). American Indian perceptions of colorectal cancer screening: viewpoints from adults under age 50. J Cancer Educ.

[14] Jones RM, Woolf SH, Cunningham TD, Johnson RE, Krist AH, Rothemich SF, Vernon SW (2010). The relative importance of patient-reported barriers to colorectal cancer screening. Am J Prev Med.

[15] McLachlan SA, Clements A, Austoker J (2012). Patients’ experiences and reported barriers to colonoscopy in the screening context–a systematic review of the literature. Patient Educ Couns.

[16] Fernandez ME, Wippold R, Torres-Vigil I, Byrd T, Freeberg D, Bains Y, Guajardo J, Coughlin SS, Vernon SW (2008). Colorectal cancer screening among Latinos from U.S. cities along the Texas-Mexico border. Cancer Causes Control.

[17] US Preventative Services Task Force (2008). Screening for colorectal cancer: U.S. Preventive Services Task Force Recommendation Statement. Ann Intern Med.

[18] Meyer JE, Narang T, Schnoll-Sussman FH, Pochapin MB, Christos PJ, Sherr DL (2010). Increasing incidence of rectal cancer in patients aged younger than 40 years: an analysis of the surveillance, epidemiology, and end results database. Cancer.

[19] Siegel RL, Jemal A, Ward EM (2009). Increase in incidence of colorectal cancer among young men and women in the United States. Cancer Epidemiol Biomarkers Prev.

[20] Ogden C, Carroll M, Curtin L, Lamb M, Flegal K (2010). Prevalence of High Body Mass Index in US Children and Adolescents, 2007–2008. JAMA.

[21] Ha EJ, Caine-Bish N (2009). Effect of nutrition intervention using a general nutrition course for promoting fruit and vegetable consumption among college students. J Nutr Educ Behav.

[22] Levy BT, Daly JM, Xu Y, Ely JW (2012). Mailed fecal immunochemical tests plus educational materials to improve colon cancer screening rates in Iowa Research Network (IRENE) practices. J Am Board Fam Med.

[23] Meade C, McKinney W, Barnas G (1994). Educating patients with limited literacy skills: the effectiveness of printed and videotaped methods about colon cancer. Am J Public Health.

[24] Houts PS, Doak CC, Doak LG, Loscalzo MJ (2006). The role of pictures in improving health communication: a review of research on attention, comprehension, recall, and adherence. Patient Educ Couns.

[25] Moseley D, Higgins S, Bramald R, Hardman F, Miller J, Mroz M, Tse H, Newton D, Thompson I, Williamson J, Halligan J, Bramald S, Newton L, Tymms P, Henderson B, Stout J (1999). Ways forward with ICT: effective pedagogy using information and communications technology in literacy and numeracy in primary schools.

[26] Moseley D, Baumfield V, Elliott J, Gregson M, Higgins S, Miller J, Newton D (2005). Frameworks for thinking: a handbook for teaching and learning.

[27] von Wagner C, Steptoe A, Wolf MS, Wardle J (2009). Health literacy and health actions: a review and a framework from health psychology. Health Educ Behav.

[28] Redwood D, Provost E, Asay E, Ferguson J, Muller J (2013). Giant inflatable colon and community knowledge, intention, and social support for colorectal cancer screening. Prev Chronic Dis.

[29] Chen E-L (2004). A Review of Learning Theories from Visual Literacy. J Educ Comp Design Online Learn.

[30] Coronado G, Farias A, Thompson B, Godina R, Oderkirk W (2006). Attitudes and beliefs about colorectal cancer among Mexican Americans in communities along the US-Mexico border. Ethn Dis.

[31] Moralez E, Rao S, Livaudais J, Thompson B (2012). Improving knowledge and screening for colorectal cancer among Hispanics: Overcoming barriers through a PROMOTORA-led home-based educational intervention. J Cancer Educ.

[32] Sanderson P, Weinstein N, Teufel-Shone N, Martinez M (2011). Assessing Colorectal Cancer Screening Knowledge at Tribal Fairs. Prev Chronic Dis.

[33] Garcia-Retamero R, Cokely E (2011). Effective communication of risks to young adults: using message framing and visual aids to increase condom use and STD screening. J Exp Psychol Appl.

[34] Leahey TM, Gokee LaRose J, Fava JL, Wing RR (2011). Social influences are associated with BMI and weight loss intentions in young adults. Obesity.

[35] Ritvo P, Myers R, Paszat L, Serenity M, Perez D, Rabeneck L (2013). Gender differences in attitudes impeding colorectal cancer screening. BMC Public Health.

[36] Katz ML, Fisher JL, Fleming K, Paskett ED (2012). Patient activation increases colorectal cancer screening rates: a randomized trial among low-income minority patients. Cancer Epidemiol Biomarkers Prev.

[37] Gonzales M, Nelson H, Rhyne RL, Stone SN, Hoffman RM (2012). Surveillance of Colorectal Cancer Screening in New Mexico Hispanics and Non-Hispanic Whites. J Community Health.

[38] Dreier M, Borutta B, Seidel G, Kreusel I, Toppich J, Bitzer E, Dierks M-L, Walter U (2013). Development of a comprehensive list of criteria for evaluating consumer education materials on colorectal cancer screening. BMC Public Health.

[39] Kentucky Cancer Program (2013). Kentucky is blue about colon cancer.

[40] New Mexico Colorectal Cancer Program (2013). Strollin’ Colon.

[41] Hart A, Barone T, Mayberry J (1997). Increasing compliance with colorectal cancer screening: the development of effective health education. Health Educ Res.

[42] National Cancer Institute (2013). Surveillance Epidemiology and End Results: Stat Fact Sheets Colon and Rectum.

[43] Ostlin P, Eckermann E, Mishra US, Nkowane M, Wallstam E (2006). Gender and health promotion: a multisectoral policy approach. Health Promot Int.

[CR44] The pre-publication history for this paper can be accessed here:http://www.biomedcentral.com/1471-2407/14/626/prepub

