# Supplementary material for: Evaluation of the walk-through inflatable colon as a colorectal cancer education tool: results from a pre and post research design
Source: BMC Cancer. 2014 Aug 28;14:626. doi: 10.1186/1471-2407-14-626 (PMC4158036; doi:10.1186/1471-2407-14-626)
Supplement: Supplementary file 1 — Additional file 1: Pre- and Post-ICAS. (PDF 704 KB) [file 12885_2013_4808_MOESM1_ESM.pdf]

☐ Obtained Colorectal Cancer Risk Assessment ☐ Received Copy of Risk Assessment

Location of Event: ________________ Date of Event:__________ Participant ID#: _________________

By answering this entrance survey, you are agreeing to participate in a research study that will help us understand how effective the inflatable colon is at teaching people about colorectal cancer. Completing this survey is optional and NOT required for entrance into the inflatable colon. You can also skip any questions you are not comfortable answering.

Please answer the following questions **BEFORE** touring the inflatable colon.

		Yes	No
1.	Have you ever obtained colorectal cancer information from the NMSU Colorectal Cancer Education Project/Booth?	☐	☐
2.	Have you ever toured the Inflatable Colon?	☐	☐

Colorectal Cancer Questions

		Yes	No
3.	Do you know what colorectal cancer is?	☐	☐
4.	Do you know what a colon polyp is?	☐	☐
5.	Have you ever talked to your doctor about colorectal cancer?	☐	☐
6.	Do you know what a cancer screening test is?	☐	☐
7.	Do you know the different types of screening tests available for colorectal cancer?	☐	☐
8.	Do you know what the following tests are:		
	Fecal Occult Blood Test (FOBT)/ Stool Blood Test?	☐	☐
	Colonoscopy?	☐	☐
	Sigmoidoscopy?	☐	☐
9.	Do you know where you can obtain screening tests for colorectal cancer?	☐	☐
10.	Do you think a diet **low in fat** and **high in fiber** helps **decrease** the risk for developing colorectal cancer?	☐	☐

Health Education Project NM STATE Cancer Health Disparities Building Bridges to Enhance Partnerships NMSU & FHCRC

☐ Obtained Colorectal Cancer Risk Assessment ☐ Received Copy of Risk Assessment

		Yes	No
11.	Do you think physical activity **decreases** the risk of developing colorectal cancer?	☐	☐
12.	Do you think the risks for developing colorectal cancer **increases** after the age of 50?	☐	☐
13.	Do you think most patients survive colorectal cancer if it is found early and removed?	☐	☐
14.	Do you think you ONLY need colorectal cancer screening if you are having any symptoms?	☐	☐
15.	Do you think that herbs and natural remedies can cure or prevent cancer?	☐	☐

		Very Unknowledgeable	Somewhat Unknowledgeable	Neutral	Somewhat Knowledgeable	Very Knowledgeable
16.	My knowledge about colorectal cancer **BEFORE** touring the inflatable colon:	☐	☐	☐	☐	☐
17.	My knowledge about how colorectal cancer progresses **BEFORE** touring the inflatable colon:	☐	☐	☐	☐	☐

		Very Likely	Somewhat Likely	Neutral	Somewhat Unlikely	Very Unlikely	Not Applicable
18.	How likely are you to get screened for colorectal cancer?	☐	☐	☐	☐	☐	☐
19.	How likely are you to talk about colorectal cancer with your:						
	Parents	☐	☐	☐	☐	☐	☐
	Grandparents	☐	☐	☐	☐	☐	☐
	Relatives (aunts, uncles, cousins)	☐	☐	☐	☐	☐	☐
	Peers (friends, colleagues, etc.)	☐	☐	☐	☐	☐	☐
	Community members	☐	☐	☐	☐	☐	☐
	Individuals at risk (50+ years of age, family history, etc.)	☐	☐	☐	☐	☐	☐

ABOUT YOU

20. What is your gender? ☐ Male ☐ Female

21. What is your age group?

☐ 20-24	☐ 25-29	☐ 30-34	☐ 35-39
☐ 40-44	☐ 45-49	☐ 50-54	☐ 55-59
☐ 60-64	☐ 65-69	☐ 65-69	☐ 70-75
☐ 75 and older			

22. How many years of school have you completed?
    - ☐ 4th grade or less
    - ☐ 5th grade through 8th grade
    - ☐ 9th grade through 12th grade, no diploma
    - ☐ High school graduate or GED
    - ☐ Some college, but no degree
    - ☐ College degree
    - ☐ Advanced degree (MD, PhD, JD, Master Degree)

23. What is your race? [CHECK ALL THAT APPLY]
    - ☐ Non-Hispanic White
    - ☐ Hispanic, Latino, Hispanic origin
    - ☐ Black or African-American
    - ☐ American Indian or Native American
    - ☐ Asian or Pacific Islander
    - ☐ Other → Specify: ______________________________

24. Do you have a health clinic where you usually go for health care?
   - ☐ Yes → [IF YES:] Where do you go? ________________________________
   - ☐ No

25. Do you have a regular doctor who you usually see for health care?
   - ☐ Yes → [IF YES:] Where do you go? ________________________________
   - ☐ No

26. Do you have any of the following health care plans?
   - ☐ Private health insurance (self paid)
   - ☐ Private health insurance (through employer)
   - ☐ Medicare
   - ☐ Medicaid
   - ☐ Veterans Affairs health insurance
   - ☐ None
   - ☐ Other → Specify: ________________________________

27. Has your doctor ever recommended one of the following colorectal cancer screening tests:

   (a) Fecal Occult Blood Test (FOBT)/ Stool Test
   - ☐ Yes → [IF YES:] When? ________________________________
   - ☐ No

   (b) Sigmoidoscopy
   - ☐ Yes → [IF YES:] When? ________________________________
   - ☐ No

   (c) Colonoscopy
   - ☐ Yes → [IF YES:] When? ________________________________
   - ☐ No

☐ Obtained Colorectal Cancer Risk Assessment ☐ Received Copy of Risk Assessment

Location of Event: ________________ Date of Event:__________ Participant ID#: _________________

By answering this exit survey, you are agreeing to participate in a research study that will help us understand how effective the inflatable colon is at teaching people about colorectal cancer. Completing this survey is optional and NOT required for entrance into the inflatable colon. You can also skip any questions you are not comfortable answering.

Please answer the following questions **AFTER** touring the inflatable colon.

Colorectal Cancer Questions

		Yes	No
1.	Do you know what colorectal cancer is?	☐	☐
2.	Do you know what a colon polyp is?	☐	☐
3.	Do you know what a cancer screening test is?	☐	☐
4.	Do you know the different types of screening tests available for colorectal cancer?	☐	☐
5.	Do you know what the following tests are:		
	Fecal Occult Blood Test (FOBT)/ Stool Blood Test?	☐	☐
	Colonoscopy?	☐	☐
	Sigmoidoscopy?	☐	☐
6.	Do you know where you can obtain screening tests for colorectal cancer?	☐	☐
7.	Do you think a diet low in fat and high in fiber helps decrease the risk for developing colorectal cancer?	☐	☐

☐ Obtained Colorectal Cancer Risk Assessment ☐ Received Copy of Risk Assessment

		Yes	No
8.	Do you think physical activity decreases the risk of developing colorectal cancer?	☐	☐
9.	Do you think the risks for developing colorectal cancer increases after the age of 50?	☐	☐
10.	Do you think most patients survive colorectal cancer if it is found early and removed?	☐	☐
11.	Do you think you ONLY need colorectal cancer screening if you are having any symptoms?	☐	☐
12.	Do you think that herbs and natural remedies can cure or prevent cancer?	☐	☐

		Very Unknowledgeable	Somewhat Unknowledgeable	Neutral	Somewhat Knowledgeable	Very Knowledgeable
13.	My knowledge about colorectal cancer **AFTER** touring the inflatable colon:	☐	☐	☐	☐	☐
14.	My knowledge about how colorectal cancer progresses **AFTER** touring the inflatable colon:	☐	☐	☐	☐	☐

		Very Likely	Somewhat Likely	Neutral	Somewhat Unlikely	Very Unlikely
15.	After touring the inflatable colon, how likely are you to get screened for colorectal cancer in the future?	☐	☐	☐	☐	☐
16.	After touring the inflatable colon, how likely are you to talk to your doctor about colorectal cancer in the future?	☐	☐	☐	☐	☐
17.	How likely is it that the inflatable colon will be accepted in your culture as an educational tool for colorectal cancer?	☐	☐	☐	☐	☐

☐ Obtained Colorectal Cancer Risk Assessment ☐ Received Copy of Risk Assessment

		Very Likely	Likely	Neutral	Unlikely	Very Unlikely	Not Applicable
18.	After touring the inflatable colon, how likely are you to talk about colorectal cancer with your:						
	Parents	☐	☐	☐	☐	☐	☐
	Grandparents	☐	☐	☐	☐	☐	☐
	Relatives (aunts, uncles, cousins)	☐	☐	☐	☐	☐	☐
	Peers (friends, colleagues, etc.)	☐	☐	☐	☐	☐	☐
	Community members	☐	☐	☐	☐	☐	☐
	Individuals at risk (50+ years of age, family history, etc.)	☐	☐	☐	☐	☐	☐
19.	After touring the inflatable colon, how likely are you to encourage others to take a tour of the inflatable colon?						
	Parents	☐	☐	☐	☐	☐	☐
	Grandparents	☐	☐	☐	☐	☐	☐
	Relatives (aunts, uncles, cousins)	☐	☐	☐	☐	☐	☐
	Peers (friends, colleagues, etc.)	☐	☐	☐	☐	☐	☐
	Community members	☐	☐	☐	☐	☐	☐
	Individuals at risk (50+ years of age, family history, etc.)	☐	☐	☐	☐	☐	☐

		Very Effective	Effective	Neutral	Ineffective	Very Ineffective
20.	How effective is the inflatable colon as an educational tool to teach people about colorectal cancer?	☐	☐	☐	☐	☐

		Very Positive	Positive	Neutral	Negative	Very Negative
21.	What is your overall reaction towards the inflatable colon?	☐	☐	☐	☐	☐
